# Eutherian-Specific Functions of BetaM Acquired through *Atp1b4* Gene Co-Option in the Regulation of MyoD Expression

**DOI:** 10.3390/life13020414

**Published:** 2023-02-02

**Authors:** Nisar Ahmad, Ivana L. de la Serna, Himangi G. Marathe, Xiaoming Fan, Prabhatchandra Dube, Shungang Zhang, Steven T. Haller, David J. Kennedy, Nikolay B. Pestov, Nikolai N. Modyanov

**Affiliations:** 1 Department of Physiology and Pharmacology, Center for Diabetes and Endocrine Research, College of Medicine and Life Sciences, The University of Toledo, Toledo, OH 43614, USA; 2 Department of Cell and Cancer Biology, College of Medicine and Life Sciences, The University of Toledo, Toledo, OH 43614, USA; 3 Department of Medicine, College of Medicine and Life Sciences, The University of Toledo, Toledo, OH 43614, USA

**Keywords:** ATP1B4, gene cooption, placental mammal evolution, muscle development, MyoD

## Abstract

Vertebrate *ATP1B4* genes represent a rare instance of orthologous gene co-option, resulting in radically different functions of the encoded BetaM proteins. In lower vertebrates, BetaM is a Na, K-ATPase β-subunit that is a component of ion pumps in the plasma membrane. In placental mammals, BetaM lost its ancestral role and, through structural alterations of the N-terminal domain, became a skeletal and cardiac muscle-specific protein of the inner nuclear membrane, highly expressed during late fetal and early postnatal development. We previously determined that BetaM directly interacts with the transcriptional co-regulator SKI-interacting protein (SKIP) and is implicated in the regulation of gene expression. This prompted us to investigate a potential role for BetaM in the regulation of muscle-specific gene expression in neonatal skeletal muscle and cultured C2C12 myoblasts. We found that BetaM can stimulate expression of the muscle regulatory factor (MRF), MyoD, independently of SKIP. BetaM binds to the distal regulatory region (DRR) of MyoD, promotes epigenetic changes associated with activation of transcription, and recruits the SWI/SNF chromatin remodeling subunit, BRG1. These results indicate that eutherian BetaM regulates muscle gene expression by promoting changes in chromatin structure. These evolutionarily acquired new functions of BetaM might be very essential and provide evolutionary advantages to placental mammals.

## 1. Introduction

Change in gene function by gene co-option is one of the key mechanisms of molecular evolution [1]. Ancestral genes can be co-opted for new functions by changes in the protein-coding and/or regulatory sequences. We demonstrated that *ATP1B4* genes, which are members of the X,K-ATPase β-subunit gene family (X = Na or H), represent an instance of orthologous vertebrate gene co-option creating fundamental changes in functional properties of the encoded BetaM proteins [2].

Evolutionary changes in BetaM function are associated with alterations in protein structure. In lower vertebrates such as fishes, amphibians, and birds, BetaM proteins are genuine Na, K-ATPase β-subunits that assemble with α-subunits into functional ion pumps in the plasma membrane [3]. In eutharians (placental mammals), the BetaM protein acquired new properties through radical changes in the structure of its N-terminal domain, with the addition of two extended Glu-rich clusters and an N-terminal Arg-rich nonapeptide, while retaining all the structural features and signature motifs specific to X,K-ATPase β-subunits [4,5,6]. As a result of these evolutionary alterations, eutherian BetaM completely lost its ancestral function as a Na, K-ATPase subunit [5]. Instead of the plasma membrane, it resides in intracellular stores, primarily in the inner nuclear membrane, thereby exposing the long N-terminal domain to the nucleoplasm [6]. Notably, the Arg-rich N-terminus of the nucleoplasmic domain is similar to nuclear localization signals and the Glu-rich clusters are considered homopolymeric amino acid repeats. The latter usually form intrinsically disordered domains, serving as flexible molecular recognition elements in many signaling proteins and transcriptional regulators [7,8,9]. 

Several lines of evidence suggest that eutherian BetaM has an important role in muscle development. We previously showed that expression of the eutherian BetaM is highly enriched in skeletal muscle and expressed at a lower level in cardiac myocytes [5,10,11]. BetaM is developmentally regulated, being the highest in myocytes during late fetal and early postnatal development [6]. The first wave of muscle development (E10.5–E14.5), when primary myotubes are formed, appears to be completed in the absence of BetaM. Robust expression of BetaM occurs on E16.5, just after the onset of secondary myogenesis, during which primary myotubes serve as a scaffold for the attachment and fusion of myoblasts into secondary myotubes, leading to growth and maturation of muscle fibers. It should be noted that this stage of in vivo skeletal muscle development is not adequately reproduced in cell culture models in vitro. Moreover, we have observed that BetaM protein disappears in primary rat myocytes after 24 h in culture. This indicates that BetaM is expressed only in myocytes within intact skeletal tissue. Presumably, BetaM expression is under strict control by extra-cellular cues that are lost in cell culture conditions.

BetaM is localized to the nuclear membrane in neonatal skeletal muscle and directly interacts with nuclear transcriptional co-regulator Ski-interacting protein (SKIP) [2]. Through this association, BetaM counter-acts SKIP to up-regulate gene expression of Smad7, a potent inhibitor of the TGF-β signaling pathway [12,13]. These findings indicate that eutherian BetaM functions as a transcriptional regulator, specifically during a critical period of perinatal development [2]. Interestingly, SKIP was implicated in the regulation of myogenesis. One study showed that SKIP interacts with poly(A)-binding protein 2 (PABP2) to increase the expression and activity of the muscle regulator factor (MRF), MyoD [14]. These prior studies led to the hypothesis that BetaM has a role in the regulation of muscle gene expression.

The MRFs are helix loop helix transcription factors, critical for commitment of mesodermal progenitors to the myocyte lineage and for myoblast differentiation into myocytes [15]. Among the MRFs, MyoD is expressed early during embryonic muscle development and plays a key role in the commitment of progenitors to the myogenic lineage [16]. High expression of MyoD continues until birth, is down-regulated in adult skeletal muscle, then re-activated in satellite cells during muscle regeneration [17,18]. MyoD expression is tightly regulated by two distal enhancer elements, which include the core enhancer (CE) and the distal regulatory region (DRR), combined with the proximal regulatory region (PRR), which is positioned close to the transcriptional start site [16]. The activity of these regions is regulated by the coordination of a number of transcriptional activators and repressors [19,20,21,22,23,24]. Epigenetic mechanisms including DNA methylation [25] and chromatin modifications also play an important role in the regulation of MyoD expression [26,27].

In this study, we investigated the requirement for BetaM in neonatal muscle and cultured myoblasts. We found that BetaM promotes the transcriptional activation of MyoD in C2C12 myoblasts. Chromatin immunoprecipitation assays in neonatal skeletal muscle and C2C12 cells indicate that BetaM occupies the DRR of the MyoD upstream enhancer and can promote epigenetic changes associated with active transcription as well as recruitment of the SWI/SNF subunit, BRG1. Taken together, these results strongly demonstrate that a co-opted function for eutharian BetaM involves epigenetic regulation of gene expression in muscle.

## 2. Materials and Methods

### 2.1. Reagents and Animals

All cell culture reagents and media were purchased from Invitrogen Corp. (Carlsbad, CA, USA) and chemicals were from Sigma-Aldrich Corp. (St. Louis, MO, USA), unless otherwise mentioned. Skeletal muscle were collected from neonatal rats, as previously described [6]. Animal procedures were conducted in compliance with federal and institutional guidelines and were approved by the Institutional Animal Care and Use Committee.

### 2.2. Cell Culture and Transfections

The BetaM, SKIP, and the Na, K-ATPase β1-subunit (NKβ1) plasmids are described in [2]. C2C12 cells were obtained from ATCC (Manassas, VA, USA) and grown in DMEM (Invitrogen, Carlsbad, CA, USA) supplemented with 10% fetal bovine serum (FBS) and 1% penicillin/streptomycin. The C2C12 cells were seeded at 1.0 × 10^5^ cells per 6 cm dish in DMEM medium supplemented with 10% FBS. Cells were transfected after 14 h with control empty vector or BetaM, SKIP, Na, K-ATPase subunit cDNA [2] using Lipofectamine 2000 according to the manufacturer’s instructions (Invitrogen, Carlsbad, CA, USA) in serum-free Opti-MEM media. After 6 h of incubation, Opti-MEM was replaced with DMEM containing 10% FBS plus antibiotics (1% penicillin/streptomycin) and maintained for a period of 40 h. Thereafter, cell lysates, or mRNA, were collected and stored at −80 °C until assayed.

### 2.3. Protein Isolation and Western Blotting

Total protein was isolated in RIPA buffer (50 mM Tris-HCl,pH 7.5, 0.1% Triton-X-100, 0.5% Nonidet P-40, 0.15 M NaCl, 1 mM EDTA, 1 mM Na_3_VO_4_, 0.5 mM PMSF, 100 μL protease inhibitor (Sigma, St. Louis, MO, USA) [2]. Concentrations in cell lysates were measured by BCA protein assay (Pierce Biotechnology, Rockford, IL, USA) according to the manufacturer’s instructions. Equal amounts of protein (as determined by BCA) were loaded and subjected to electrophoresis on 10% SDS-PAGE gels then transferred to PVDF membranes, exactly as described in [2]. After blocking for non-specific binding with 5% nonfat milk, the membranes were washed and incubated with primary anti-BetaM antibody [11] (1:750 dilution), anti-SKIP antibody (1:500 dilution)) [28], or anti MyoD antibody (C-20): sc-304 (1:200 dilution; Santa Cruz Biotechnology Inc., Santa Cruz, CA, USA) for one hour, washed twice followed by addition of HRP-linked secondary antibody (1:10,000 dilution; Santa Cruz Biotechnology Inc., Santa Cruz, CA, USA) for two hours. The membranes were washed and the respective protein bands were visualized using Lumigen TMA-6 solutions A and B (Amersham Biosciences, Amshem, UK). MyoD bands were quantified by densitometry using Image Quant software version 5.2, as we performed previously in [2].

### 2.4. RNA Extraction and Reverse-Transcription-PCR (RT-PCR)

Trizol reagent (Invitrogen Carlsbad, CA, USA) was used according to the manufacturer’s directions to extract total RNA from the cells. The RNA was further purified by using DNA free reagent (Ambion Inc., Austin, TX, USA) and quantitated using a spectrophotometer. Total RNA (1 µg) from each sample was reverse-transcribed using Superscript™ First-Strand Synthesis System for RT-PCR (Invitrogen, Carlsbad, CA, USA) as per manufacturer’s instructions. The cDNA was amplified to measure the expression levels of MyoD, BetaM, SKIP, and GAPDH by using the following primer sets:

MyoD:
Forward: GCA GGC TCT GCT GCG CGA CC
Reverse: TGT AAT CCA TCA TGC CAT

BetaM:
Forward: GAG CTT GGA GAT CCT GTG AAG G
Reverse: GGA GGT CAA AAG AAG CCG ACT

SKIP
Forward: TGA CCA AAG GCT CTT CAA CCA
Reverse: GCC ATA TCT TTC CCA CCT CTC C

GAPDH:
Forward: TGC ACC ACC AAC TGC TTA G
Reverse: GAG GCA GGG ATG ATG TTC

PCR amplifications were carried out using the following conditions: 95 °C, 1.5 min; followed by 30 cycles at 94 °C, 40 s, 55°C 40 s, and 72 °C 1 min. Mouse GAPDH was amplified at the same time as an internal control. Equal volumes of PCR product from each group were electrophoresed on 1.5% (*w/v*) agarose gels and visualized with ethidium bromide. Bands were quantified by densitometry using Image J software.

### 2.5. Luciferase Assays

The PGL2 luciferase reporter vectors containing the MyoD promoter, MyoD PRR, MyoD DRR, and MyoD CE are described in detail in [29]. Renilla plasmid (Renilla Luciferase Assay System, Promega, Madison, WI, USA) was used as an internal control. For transfections, 2 × 10^6^ C2C12 cells were allowed to attach overnight, medium was replaced with Opti-MEM, and the following plasmids were transfected: (0.25 µg) MyoD, MyoD PR, MyoD CE, MyoD DRR (0.04 µg) Renilla, (0.5 µg) β-m, and SKIIP, (0.25 µg) using 2 µL of lipofectamine (Invitrogen, Carlsbad, CA, USA) in 100 µL of serum-free medium. Lipofectamine-DNA binding was allowed to proceed for 15 min, and then, the mixture was added to the cells. After 4 h, appropriate amounts of serum were added, and the cells were allowed to grow for 48 h, scraped and washed first with PBS and then with 250 mM Tris-HCl buffer, pH 7.2. Total cell extracts were prepared by treating the cells with lysis buffer (Pierce, Rockford, Il, USA), and transactivation of MyoD luciferase constructs was determined using the luciferase kit from Pierce as per the manufacturer’s protocol. The activity of the Renilla luciferase was used for normalizing the transfection efficiency. The results presented are the average of three experiments.

### 2.6. MyoD DRR Luciferase Deletion Constructs

Deletions of E-box 4 and CArG sequences in MyoD DRR luciferase construct were introduced by using the PCR based QuickChange Kit (Stratagene, La Jolla, CA, USA) following the manufacturer’s instructions. The primers used were given under:

Ebox-4 Deletion:
Forward: GGG CAG GGT GCG TGA AGG GTT TCC AGA GGC TAT ATA TAT A
Reverse: TAT ATA TAT AGC CTC TGG AAA CCC TTC ACG CAC CCT GCC C

CArG Deletion:
Forward: CAC ATT CCT TTC CAG AGG GCA GCC AAG GGA GCT GAG AGG G
Reverse: CCC TCT CAG CTC CCT TGG CTG CCC TCT GGA AAG GAA TGT G

The Ebox-4 /CArG deleted MyoD DRR luciferase construct was constructed by two cycles of deletion. First Ebox-4 was deleted and the same construct was used for deleting CArG in the next cycle. All deletion constructs were confirmed by sequencing.

### 2.7. Chromatin Immunoprecipitation Assays (ChIP) 

ChIP assays were performed using chromatin from rat neonatal muscle (tongue) or murine C2C12 myoblasts as described [2]. The purified DNA fragments were amplified by carrying out a radioactive PCR. The amplified DNA samples were run on 7% polyacrylamide gel. The gels were dried under vacuum and exposed to phosphorimager screens overnight. The screens were scanned on a photo-documentation unit to obtain the final results. For some experiments, ChIPs were analyzed by quantitative PCR (qPCR) Quantitative PCR (qPCR) using SYBR Green master mix (Qiagen, Hilden Germany) with an Applied Biosystems 7500 PCR (Waltham, MA, USA. Antibodies used were: α-BetaM [11], α-SKIP [28], α-BRG1 (Abcam, Cambridge, UK), α-tetra-acetylated histone H4 (acH4), and α-histone H3 tri-methylated lysine 4(H3K4me3) (Active Motif, Carlsbad, CA, USA). The following are primer sets used for amplification:

Rat PRR
Forward TAG GCA CTG GAG AGA CTT GG
Reverse GCC TCA AGC CAA TAG GAG TGT AG

Rat DRR2
Forward TAG ACA CAA GCC AGC AAT GC
Reverse TAT AAA TGG AGA GCT GGC TT

Rat CE
Forward ACA TGA GCC CCA CAG CAT TTG
Reverse GAG CTA GAG AAA CCG GAG AAG A

Rat GAPDH
Forward CAT TAA CGT CAA CTA CAT GG
Reverse TGA TGA CCA GCT TCC CAT TCT CAG C

Mouse DRR
Forward GGGCTGGTCCTGTTCCACC
Reverse GCTATAAATGGAGAGCTGGCTTTT

Mouse IgH enhancer
Forward GCCGATCAGAACCAGAACACC
Reverse TGGTGGGGCTGGACAGAGTGTTTC

### 2.8. Electrophoretic Mobility Shift Assays (EMSA)

Electrophoretic mobility shift assays were performed using nuclear extracts from neonatal rat muscle [30]. The oligonucleotides used in this experiment are shown in Table 1.

### 2.9. Statistical Analysis 

Statistically significant differences between two groups were calculated by the student’s t-test. One-way ANOVA followed by post-hoc Tukey was used to analyze significant differences between more than two groups.

## 3. Results

### 3.1. Beta M Promotes Expression of the Muscle Determining Factor, MyoD in C2C12 Myoblasts

**Figure 1:**
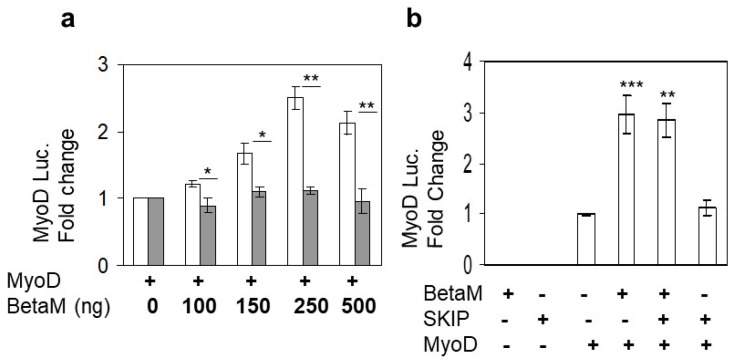
BetaM stimulates MyoD reporter activity. (**a**) Reporter activity in C2C12 cells with co-transfection of the wildtype MyoD-PGL2 reporter (contains CE, DRR, and PRR) and increasing amounts of BetaM (white bars) or as a control, NKβ1 (grey bars). (**b**) The effect of BetaM with and without SKIP on MyoD luciferase activity. Basal activities of the reporter plasmids were set to 1 and all results are shown as means ± SD, *n* = 3, * *p* < 0.05, ** *p* < 0.01, *** *p* < 0.005.

Expression of BetaM occurs on E16.5 just after the onset of secondary myogenesis, a stage of in vivo skeletal muscle development that is not adequately reproduced in cell culture models in vitro [6]. Thus, BetaM is not expressed in cell culture models such as C2C12 cells. Therefore, we exogenously expressed BetaM in C2C12 cells. Using this strategy, we previously found that BetaM associates with the nuclear transcriptional co-regulator, SKIP, a component of the TGF-β pathway [2]. In order to determine if BetaM regulates transcription of muscle genes, we co-transfected a MyoD luciferase construct and increasing amounts of BetaM, or as a control, the structurally related Na, K-ATPase β1-subunit (NK β1). BetaM (white bars) stimulated MyoD luciferase activity in a dose-dependent manner while expression of NK β1 (grey bars) had no significant effect on luciferase activity (Figure 1a). To determine if the effect of BetaM on MyoD transcription involves SKIP, we co-transfected BetaM and SKIP. BetaM stimulated luciferase activity to the same levels whether expressed alone or in combination with SKIP (Figure 1b), suggesting that the effects of BetaM on MyoD transcriptional activity are independent of its association with SKIP.

**Figure 2:**
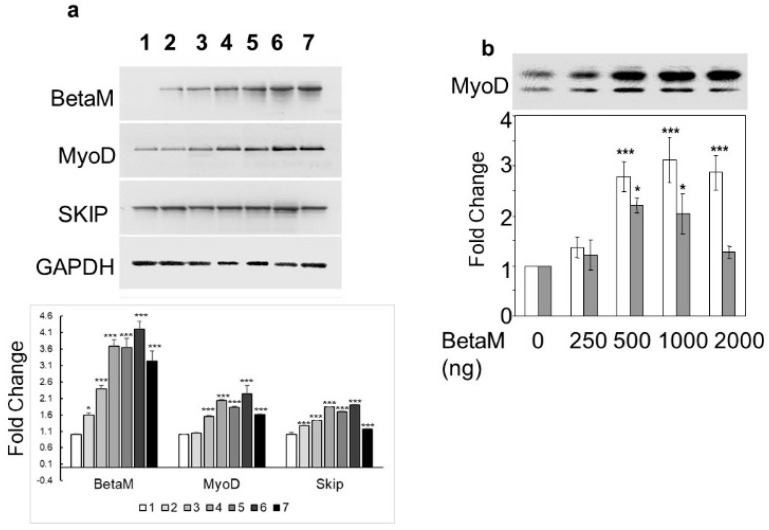
BetaM promotes endogenous MyoD expression. (**a**) Effect of BetaM on endogenous mRNA levels of MyoD, SKIP, and as a control, GAPDH. C2C12 cells were transfected with increasing amounts of BetaM (Lane: 1: 0 ng, 2: 100 ng; 3: 150 ng; 4: 200 ng; 5: 250 ng; 6: 300 ng; 7: 500 ng) and analyzed by semi-quantitative RT-PCR. Top: A representative gel is shown. Bottom: Bands were quantified by densitometry. The levels of BetaM, MyoD, and SKIP were normalized to GAPDH and are shown relative to basal levels. (**b**) Up-regulation of endogenous MyoD protein by BetaM in C2C12 cells. **Top**: A representative Western blot where equal amounts of total protein were loaded. Upper bands are hyper-phosphorylated MyoD and lower bands are hypo-phosphorylated MyoD. **Bottom**: Bands were analyzed by densitometry and normalized to the levels of basal hyper-phosphorylated and hypo-phosporylated MyoD. White bars represent hyper-phosphorylated MyoD and Grey bars represent hypo-phosphorylated MYOD. The data are from three independent experiments (means ± SE). * *p* < 0.05, *** *p* < 0.005.

To determine if BetaM could activate expression of the endogenous MyoD gene, we transfected increasing amounts of BetaM in C2C12 cells. BetaM significantly increased endogenous MyoD mRNA levels, as well as SKIP mRNA levels, in a generally dose-dependent manner (Figure 2a). We confirmed the effect of BetaM on endogenous MyoD expression by performing Western blotting. BetaM promoted a significant increase in both hypo- and hyper-phosphorylated forms of MyoD protein [31,32] (Figure 2b). These data strongly suggest that BetaM regulates MyoD expression in cultured myoblasts and that this effect may be independent of SKIP.

### 3.2. BetaM Binds to an E-Box Element and the CArG Box in the DRR Region of MyoD in Rat Skeletal Muscle

**Figure 3:**
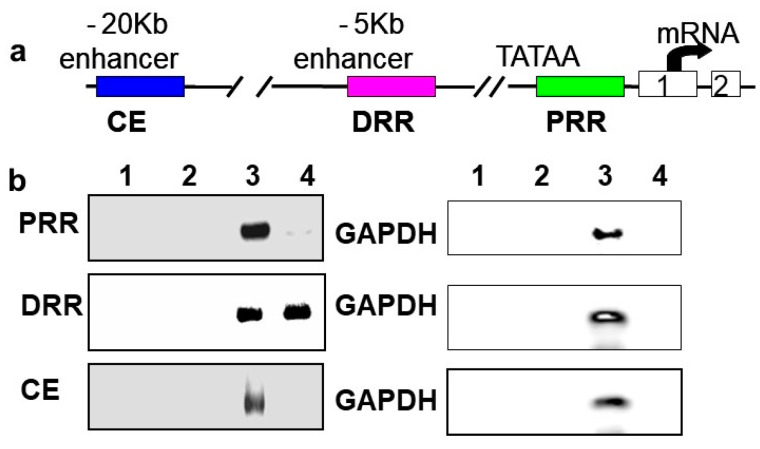
BetaM binds to the DRR of MyoD in rat skeletal muscle. (**a**) Regulatory motifs of the MyoD promoter upstream region. (**b**) ChIP assay of PRR, DRR, and CE regulatory regions using rat neonatal skeletal muscle chromatin. Controls: Chromatin alone (1), IgG (2), Input, 0.05% of total (3). ChIP with BetaM antibody (4). GAPDH was used an internal control for nonspecific or background DNA immunoprecipitation.

In vivo chromatin immunoprecipitations (ChIPs) were performed using rat neonatal muscle to validate the results in C2C12 cells and to determine if BetaM regulates MyoD expression by directly binding to a regulatory region of the MyoD locus. MyoD transcription is regulated by three important regulatory regions: proximal regulatory region (PRR), distal regulatory region (DRR), and core enhancer (CE) [33,34] 

(Figure 3a). BetaM was detected on the DRR but not on the PRR or CE of the MyoD upstream region (Figure 3b). Taken together, these in vivo results indicate that our in vitro findings are biologically relevant and suggest that BetaM binds to the MyoD DRR and directly promotes MyoD transcription.

**Figure 4:**
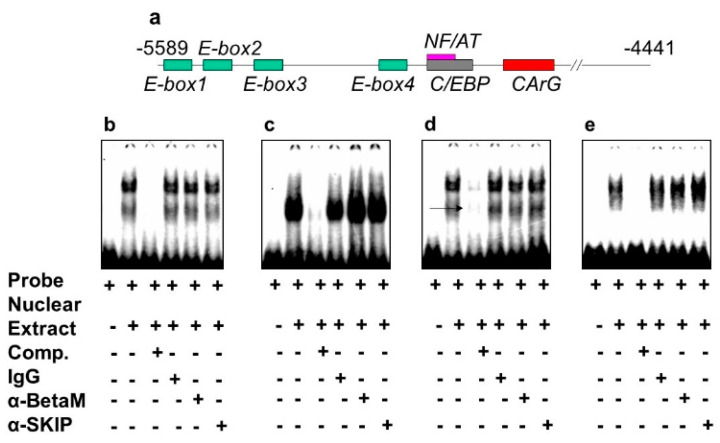
BetaM is not present on E-box 1, 2, 3 NF/AT or C/EBP elements of the rat MyoD DRR region. (**a**) Response elements in the rat MyoD DRR. Electrophoretic mobility shift assays using the following probes: (**b**) E-box1, (**c**) E-box2, (**d**) E-box3 and (**e**) NF/AT; C/EBP.

The DRR of the MyoD upstream region contains several known response elements. Among these elements, E-box 2, E-box 4, NF/AT, C/EBP, and CArG (Figure 4a) are highly conserved in human, rat, and mouse. To investigate which of these elements bind transcriptional complexes containing BetaM, we performed electrophoretic mobility shift assays (EMSA) using nuclear extract from rat neonatal muscle. As expected, these elements were bound by nuclear proteins. However, the addition of BetaM and SKIP antibodies did not produce any additional mobility changes, suggesting that BetaM and SKIP do not bind to E-boxes 1,2,3, NF/AT, or C/EBP sequences (Figure 4b–e).

**Figure 5:**
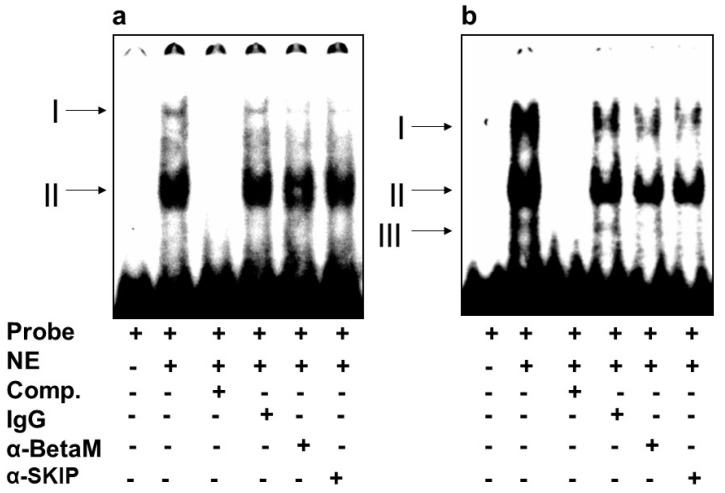
BetaM binds E-box4 and CArG elements. Electromobility mobility shift assays of (**a**) E-box4 and (**b**) CArG sequences. Arrows represent DNA–protein complexes (NE: nuclear extract, Comp: competitor).

We then investigated whether BetaM binds to E-box4 and/or the CArG box. We detected two complexes (I and II) on E-box 4, of which complex I was competed by the addition of antibodies to either BetaM or SKIP (Figure 5a). Of the complexes (I, II, III) detected on the CArG probe, we found that I and II were competed by the addition of either BetaM or SKIP antibodies (Figure 5b).

These data suggest that BetaM and SKIP bind to both E-box4 and CArG elements of the DRR. 

**Figure 6:**
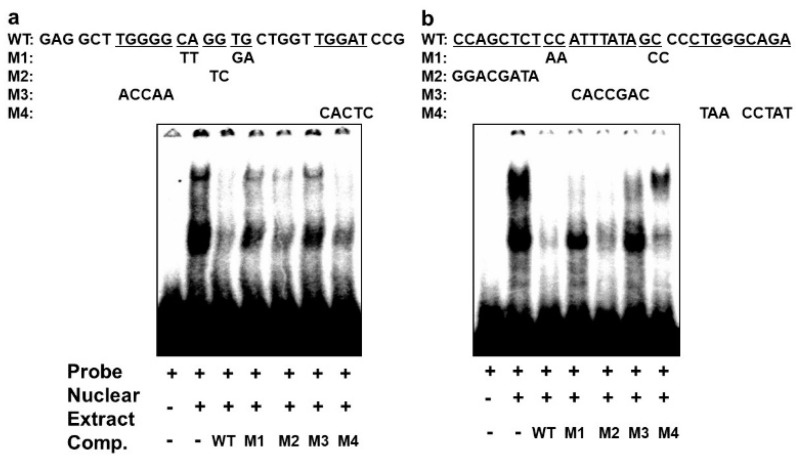
Specificity of complexes on E-box4 and CArG. Competition of electrophoretic mobility shift assays of: (**a**) E-box4 and (**b**) CArG elements using unlabeled (cold) wild type (WT) and mutant (M1 to M4) oligonucleotides. Underlined wild type (WT) sequences indicate the positions of mutations.

In order to confirm that the different complexes we had detected on the E-box 4 and the CArG box are specifically bound, we performed competition EMSAs using cold wildtype and mutant sequences. An oligonucleotide with wildtype E-box4 and one with mutations that preserved the E-box consensus (M2 and M4) effectively competed both complexes I and II, whereas oligonucleotides with disruptions in the E-box consensus did not effectively compete (M1 and M3) (Figure 6a). Similarly, an oligonucleotide with the wildtype CArG sequence effectively competed all three complexes, whereas M1 and M3 containing mutations in the CArG box did not compete with complex III, and M4 carrying mutations in the flanking region did not compete with complex I. Taken together, these data indicate that BetaM binds to E-box4 and CArG boxes in the DRR upstream of the MyoD gene.

### 3.3. Transactivation of the MyoD DRR by BetaM Requires E-Box4 and CArG Elements

**Figure 7:**
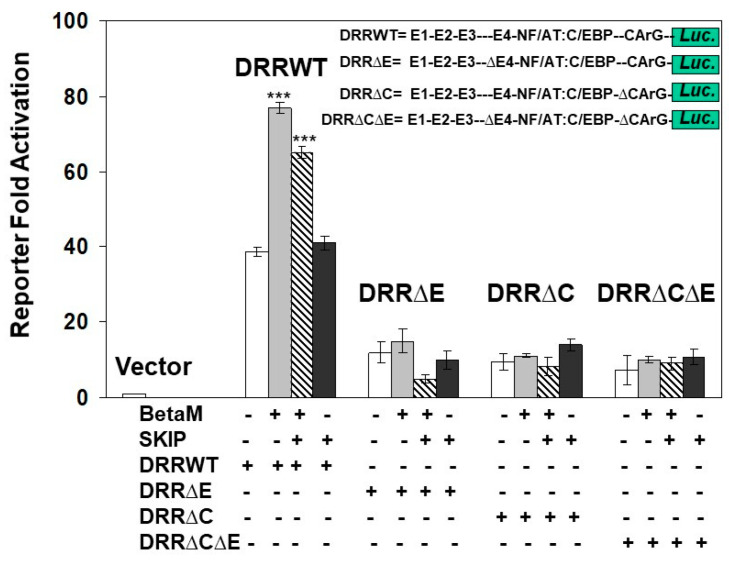
Transactivation of the DRR luciferase construct by BetaM is lost with the deletion of E-Box4 and CArG elements. C2C12 cells were co-transfected with either BetaM or SKIP and luciferase reporters driven by wild-type DRR or constructs bearing deletions of E-box 4 and CArG elements. Basal activity of the PGL2 luciferase vector was set to 1, and all results are shown as means ± SD, *n* = 3, *** *p* < 0.005.

To determine if the E-box4 and CARrG elements of the DRR are required for Beta-M mediated activation of the DRR, we performed luciferase assays with wild type DRR and deletion constructs. BetaM activated wild type DRR luciferase activity two-fold over vector control when both E-box4 and CArG elements were present but failed to activate luciferase activity when either or both were deleted (Figure 7). Interestingly, SKIP did not activate the DRR when transfected alone and slightly inhibited trans-activation by Beta-M when the two were co-transfected (Figure 7). These data demonstrate that BetaM activates MyoD expression through the E-box4 and CArG elements of the DRR in vivo and confirm that transcriptional activation by BetaM in C2C12 cells is independent of its association with SKIP.

### 3.4. BetaM Promotes BRG1 Recruitment and Histone Modifications on the DRR of the MyoD Locus

**Figure 8:**
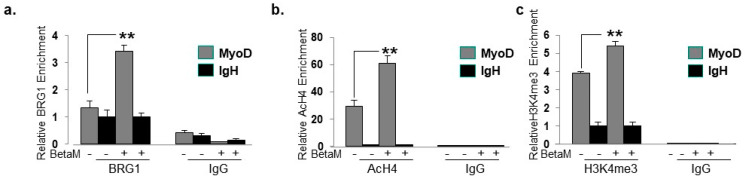
BetaM promotes BRG1 recruitment and histone modifications on the DRR of MyoD. ChIP assays were performed on control and BetaM transfected C2C12 cells using antibodies to detect (**a**) BRG1 (**b**) Tetra-acetylated histone H4 (AcH4) and (**c**) Tri-methylated histone H3 at lysine 4 (H3K4me3). IgG antibody was used as a control. Quantitative (q) PCR was performed to detect ChIP signals. Enrichment on the MyoD DRR was determined relative to that on the silent IgH enhancer. Expression of BetaM significantly enhanced recruitment of BRG1, histone H4 acetylation, and histone H3K4 tri-methylation on the MyoD promoter. The data are from three independent experiments. Results are shown as means ± SE (** *p* < 0.01).

To elucidate the mechanisms by which BetaM promotes MyoD transcription in C2C12 cells, we performed ChIPs to detect changes in co-activator recruitment and histone post-translational modifications when BetaM is expressed. The BRG1 subunit of the SWI/SNF chromatin remodeling complex plays an extensive role in myoblast proliferation and differentiation [35,36,37,38]. Our data indicate that BetaM significantly enhances BRG1 recruitment to the MyoD DRR (Figure 8a) and enhances the levels of two epigenetic marks that are associated with actively transcribed promoters: histone H4 acetylation (Figure 8b) and histone H3K4 tri-methylation (Figure 8c). Taken together, these data suggest that BetaM promotes MyoD expression by enhancing recruitment of SWI/SNF chromatin remodeling enzymes and promoting changes in chromatin structure.

## 4. Discussion

BetaM gained entirely new functions during vertebrate evolution, ceasing to assemble with α–subunits into ion pumps in the plasma membrane and instead localizing to the nuclear membrane in eutharians [6]. BetaM expression is confined primarily to skeletal and cardiac muscle, with the highest expression occurring in late fetal and early neonatal development [6]. BetaM becomes highly expressed at E16.5, just after the onset of secondary myogenesis, suggesting that BetaM is required for growth and maturation of muscle fibers.

In order to elucidate new function(s) of eutharian BetaM, we first identified potential BetaM-interacting proteins using the yeast two-hybrid system and detected SKIP as a BetaM partner in the muscle [2]. Importantly, SKIP was reported to enhance MyoD expression and activity [14]. These previous observations led us to investigate the role of BetaM in the regulation of MyoD gene expression. Due to the restricted temporal pattern of BetaM expression and lack of BetaM in cultured myocytes, we exogenously expressed BetaM to elucidate its role in C2C12 cells and validated the results with experiments in neonatal rate muscle that robustly expresses BetaM.

The findings from this study strongly support the hypothesis that BetaM activates MyoD expression in neonatal muscle and in cultured myoblasts. However, our data suggest that BetaM activates MyoD independently of SKIP. SKIP is a multi-functional protein that can act as a transcriptional activator or repressor [39]. Our prior study indicated that BetaM associates with SKIP and converts SKIP from a repressor to an activator of SMAD7 expression [2]. SMAD7 inhibits TGFβ signaling but was reported to promote myogenesis by interacting with MyoD [40]. Interestingly, although we detected both BetaM and SKIP binding on E-box4 and CArG elements of the MyoD DRR (Figure 5), we found that expression of BetaM, but not SKIP, stimulated MyoD luciferase activity (Figure 1 and Figure 7). Our findings are consistent with another study, which reported that SKIP overexpression in C2C12 cells did not affect myogenesis [41]. Therefore, BetaM activates MyoD expression through a transcriptional mechanism that is independent of its association with SKIP.

BetaM interacts with other proteins that could potentially be involved in promoting epigenetic changes required for muscle development [42]. Consistent with BetaM localization to the nuclear envelope, BetaM interacts with lamina-associated protein LAP1 and myocyte nuclear envelope protein SYNE1. Mutations in the human gene, TOR1AIP, which encodes LAP1, was linked to muscular dystrophies [43] and disruption of LAP1 in mouse skeletal muscle leads to muscular dystrophy phenotype [44]. SYNE1 is linked to Emery-Dreifuss muscular dystrophy [45]. Thus, the role of BetaM in the regulation of muscle gene expression may involve interactions with LAP1 and SYNE1. Additional studies will be required to determine the functional consequences of BetaM and LAP1 interactions. However, lamina associated proteins are attractive candidates for mediating the observed epigenetic changes induced by BetaM on the MyoD promoter. We observed that Beta-M promotes MyoD expression by recruiting the BRG1 component of the SWI/SNF chromatin complex and enhancing histone covalent modifications associated with gene activation. As lamina-associated proteins are associated with the epigenetic regulation of muscle gene expression [46,47], an intriguing hypothesis is that BetaM is involved in chromatin organization by virtue of its positioning in the nuclear envelope.

In conclusion, BetaM function radically changed during the evolution of vertebrae organisms into placental mammals through gene co-option. Instead of a subunit of the Na, K-ATPase located in the cell membrane, eutherian BetaM is a component of the nuclear membrane and regulates muscle gene expression through epigenetic mechanisms. Future work will seek to elucidate the selective pressures that promoted this evolutionary change in BetaM function.

## Figures and Tables

**Table 1:** Oligonucleotides used for electrophoretic mobility shift essays. Mutations in the oligonucleotides are shown in lower case letters.

Region	Forward	Reverse	
Ebox1	ACT GCT GTG AGA ACA TCT GAC ATC CAC CAC	GTG GTG GAT GTC AGA TGT TCT CAC AGC AGT	WT
Ebox2	CCA CCA CCT AGT TCA TTT GCC AGA CTC CCA AGG	CCT TGG GAG TCT GGC AAA TGA ACT AGG TGG TGG	WT
Ebox3	GAC CAG GAC CAC ATC TGC GCC CAG CCA CAA	TTG TGG CTG GGC GCA GAT GTG GTC CTG GTC	WT
Ebox4	GAG GCT TGG GGC AGG TGC TGG TTG GAT CCG	CGG ATC CAA CCA GCA CCT GCC CCA AGC CTC	WT
Ebox4 M1	GAG GCT TGG GGt tGG gaC TGG TTG GAT CCG	CGG ATC CAA CCA Gtc CCa aCC CCA AGC CTC	Mutant
Ebox4 M2	GAG GCT TGG GGC Atc TGC TGG TTG GAT CCG	CGG ATC CAA CCA GCA gaT GCC CCA AGC CTC	Mutant
Ebox4 M3	GAG GCT acc aaC AGG TGC TGG TTG GAT CCG	CGG ATC CAA CCA GCA CCT Gtt ggt AGC CTC	Mutant
Ebox4 M4	GAG GCT TGG GGC AGG TGC TGG Tca ctc CCG	CGG gag tgA CCA GCA CCT GCC CCA AGC CTC	Mutant
NFAT	TTG GAT CCG GTT TCC AGA GGC TAT ATA TAT AAA	TTT ATA TAT ATA GCC TCT GGA AAC CGG ATC CAA	WT
CArG	CCA GCT CTC CAT TTA TAG CCC CTG GGC AGA	TCT GCC CAG GGG CTA TAA ATG GAG AGC TGG	WT
CArG M1	CCA GCT CTa aAT TTA Tcc CCg gTG GGC AGA	TCT GCC CAc cGG ggA TAA ATt tAG AGC TGG	Mutant
CArG M2	ggA cga taC CAT TTA TAG CCC CTG GGC AGA	TCT GCC CAG GGG CTA TAA ATG Gta tcg Tcc	Mutant
CArG M3	CCA GCT CTC Cca ccg acG CCC CTG GGC AGA	TCT GCC CAG GGG Cgt cgg tgG GAG AGC TGG	Mutant
CArG M4	CCA GCT CTC CAT TTA TAG CCt aaG ccC tat	ata Ggg Ctt GGG CTA TAA ATG GAG AGC TGG	Mutant

## Data Availability

Data is available upon request.
